# The Impact of S‐220–Loaded Silk Hydrogels on In Vitro Neurite Outgrowth in 2 and 3 Dimensions

**DOI:** 10.1002/adbi.70157

**Published:** 2026-09-08

**Authors:** Hongming Ma, F. Philipp Seib, Wenlong Huang

**Affiliations:** ^1^ Institute of Medical Sciences School of Medicine Medical Sciences & Nutrition University of Aberdeen Aberdeen Scotland UK; ^2^ Pharmaceutical Technology & Biopharmaceutics Institute of Pharmacy Friedrich Schiller University Jena Jena Germany; ^3^ Strathclyde Institute of Pharmacy and Biomedical Sciences University of Strathclyde Glasgow Scotland UK

**Keywords:** *B. mori* silk hydrogel, cortical neuron, Epac2, in vitro modelling, neurite outgrowth, S‐220

## Abstract

Silk hydrogels have attracted increasing interest as biocompatible substrates for neural cell culture and regenerative research. Recent research has shown that activation of exchange protein directly activated by cAMP 2 (Epac2) promotes neurite outgrowth in several neuronal models. Here, we investigated the effects of *Bombyx mori* silk hydrogel, alone or in combination with S‐220, a non‐hydrolysable cAMP analogue that selectively activates Epac2, on neurite outgrowth. Epac2 activation significantly and dose‐dependently enhanced both the total and longest neurite length. Neurons cultured on silk hydrogel alone exhibited neurite outgrowth comparable to control neurons, indicating good biocompatibility of the hydrogel. Addition of S‐220 to the culture medium with silk hydrogel modestly improved neurite morphology; however, incorporation of S‐220 directly into the hydrogel did not enhance neurite outgrowth but instead reduced neurite length. A subsequently established three‐dimensional cortical neuron culture model using silk hydrogel supported neurite extension and increased branching complexity beyond that observed with two‐dimensional cultures, despite a shorter overall neurite length. Collectively, these findings support silk hydrogel as a permissive biomaterial substrate for in vitro cortical neuron culture. The system provides a useful platform for investigating neurite outgrowth, neuron‐biomaterial interactions and the delivery of bioactive compounds in two‐ and three‐dimensional neural cultures.

## Graphical abstract

Postnatal cortical neurons were cultured on or within 1% Bombyx mori silk hydrogel to assess neurite outgrowth in 2D and 3D. S‐220 was delivered either through the culture medium or directly within the hydrogel. Silk hydrogel supported cortical neuron culture, S‐220 in the medium enhanced neurite outgrowth, and 3D culture promoted multidirectional neurite extension and increased branching complexity.

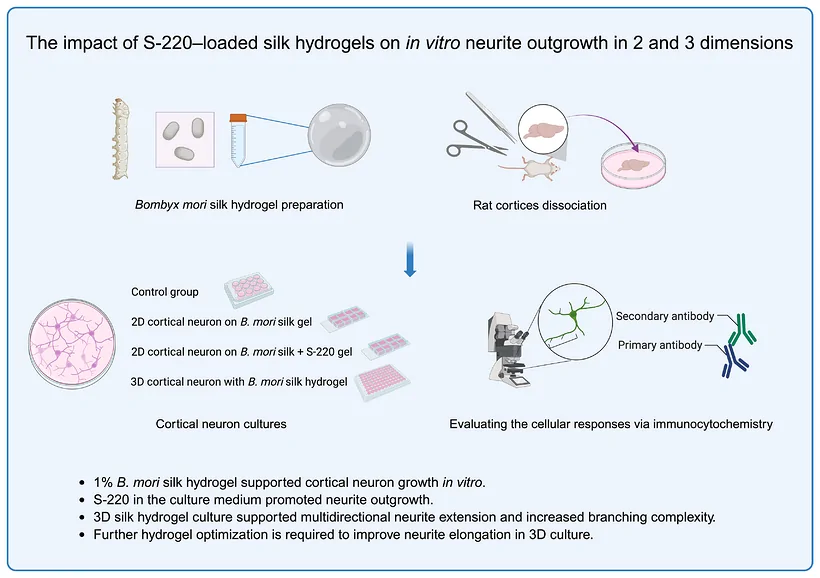

## 1 Introduction

The domesticated silkworm *Bombyx mori (B. mori)* is utilized for large‐scale commercial silk production (i.e., sericulture). *B. mori silk* fibroin has an established clinical track record in regulated medical‐device formats, including long‐standing use as sutures and surgical meshes [1]. Beyond these traditional fibrous formats, silk fibroin can be reprocessed into aqueous silk solutions. This solution can be used to produce silk films, sponges, particles, and hydrogels, making silk a versatile biomaterial platform for tissue engineering and drug delivery applications [1, 2, 3, 4, 5]. Silk‐based hydrogels are of particular interest because they are hydrated, tissue‐conformal materials that can provide a three‐dimensional (3D) microenvironment while also acting as depots for cells or bioactive molecules [2]. Direct clinical use of silk‐based injectable materials has also been reported, including silk‐hyaluronic acid formulations for vocal fold augmentation (Silk Voice), supporting the broader biomedical relevance of silk‐derived materials [6].

Silk hydrogels can be broadly classified into physically crosslinked [7] or chemically crosslinked types [8]. Physical crosslinking relies on silk gelation and the formation of cross‐linked beta‐sheets, which subsequently undergo physical cross‐linking to form hydrogels when immersed in aqueous solutions. Physically cross‐linked hydrogels can be fabricated to show structural similarities to many different tissues in the human body simply by varying the gel concentration. These silk hydrogels provide excellent biocompatibility while also possessing a ready ability to encapsulate hydrophilic drugs [2].

Physically crosslinked silk hydrogels are also well suited for injection, as they exhibit shear thinning and undergo only minimal swelling during the solution–gel transition. Of particular interest to the present study is the discovery that silk hydrogels can also be injected prior to gelation, thereby allowing a low viscosity precursor solution to be delivered minimally invasively and to percolate through irregular microcavities and interstitial spaces before gelation, achieving in situ conformal filling and stabilized retention at the target site, an advantage that is particularly relevant for the complex lesion architect [3, 4, 5]. This strategy is also appealing within the complex spinal cord injury lesion architecture, including cystic cavities and perilesional tissue.

Spinal cord injury affects around 27 million people worldwide and results in about 900,000 new cases annually [9]. These injuries, which often lead to irreversible neurological deficits, currently have no curative treatment and impose considerable societal and economic burdens. Repair is hindered by multiple factors, including the poor regenerative potential of neurons in the adult mammalian central nervous system (CNS), the development of post‐injury cavitation and glial scarring, and the presence of an inhibitory molecular milieu within the lesion environment. Applications of single approaches for spinal cord repair, such as employing an implant to bridge the cavity and promote nerve reconnection or utilizing chemical cues to prompt axonal regrowth, have not yet manifested as being clinically feasible. A combinatorial approach is viewed as more likely to yield success, as merging different strategies may generate additive or synergistic effects to more effectively address the multiple components contributing to the intricate nature of spinal cord injuries [10]. To develop and optimize such strategies, in vitro neuron culture models are widely used to interrogate neuronal survival, neurite extension, and responses to candidate biomaterials and bioactive cues under controlled conditions, providing a tractable platform for mechanistic studies and pre‐screening prior to in vivo evaluation.

One key method used in neuroscience research is primary cortical neuron culture. Cortical neurons can be freshly isolated from living brains and then cultured [11]; these neurons are histologically, biologically and genetically very similar to in vivo neurons. Furthermore, because they are derived from the cortex, they represent an in vitro model expected to display repair processes close to those involved in the CNS itself. Compared with in vivo CNS models, in vitro cortical neuron culture has the advantages of easy sampling, high sample uniformity, relatively constant and controllable experimental conditions, easy observation of detection indicators and the ability to effectively eliminate the influence of many interfering factors [12]. In addition, in vitro cultures can significantly reduce the number of experimental animals required per experiment, as well as their suffering.

Primary cortical neuron cultures provide a tractable and physiologically relevant in vitro platform to interrogate neuronal survival and neurite extension under well controlled conditions, and they are widely used to pre‐screen candidate biomaterials and bioactive cues prior to in vivo testing. In this study, postnatal rat cortical neurons were selected because primary neurons from early postnatal tissue are most commonly used in long term culture paradigms and offer robust, reproducible neuronal outgrowth assays suitable for mechanistic comparisons [13]. 3D cortical culture systems have further improved the physiological relevance of in vitro neural models by allowing neurons to grow within spatially complex matrices rather than on flat two‐dimension (2D) substrates. Previous work has demonstrated that silk‐based and extracellular matrix enriched systems can support 3D cortical neuron organization and function. For example, Tang‐Schomer et al. developed a bioengineered functional brain like cortical tissue model using a silk protein scaffold combined with extracellular matrix components [14], while Chwalek et al. described a silk‐collagen‐based protocol for generating 3D cortical brain tissue constructs seeded with primary cortical neurons [15]. Other studies have shown that extracellular matrix composition can influence neuronal network formation and functional activity in 3D cortical tissue models. These findings highlight the importance of scaffold architecture, matrix composition and culture dimensionality in shaping neuronal morphology and connectivity.

We focus on Epac2, a neuronal enriched cAMP effector implicated in the regulation of neuronal differentiation and neurite growth, making it a biologically plausible target for pro‐regenerative signaling in CNS repair contexts. To selectively engage this pathway, we employed S‐220, a cAMP analogue reported to act as an Epac2 selective agonist. Our previous research found that S‐220 can reduce PKA activation relative to cAMP, enabling a more isoform specific interrogation than broadly elevating intracellular cAMP [16, 17]. We therefore investigated the effects of silk hydrogels, alone or combined with S‐220, on neurite outgrowth and neuronal survival in a postnatal cortical neuron model. The aim of this study was to evaluate 1% *B. mori* silk hydrogel, as a substrate for cortical neuron culture in 2D and 3D, to determine how selective Epac2 activation influenced neurite outgrowth when S‐220 was supplied in the culture medium or incorporated directly into the hydrogel. This approach allowed us to assess neuronal responses to the silk hydrogel environment and to compare different strategies for delivering a neurite growth‐promoting compound in vitro.

## 2 Materials and Methods

### 2.1 Production of a Cortical Neuron Population

All procedures involving the use of live animals and animal tissues were performed in accordance with the 1986 UK Home Office (Scientific Procedures) Act and were approved by the Animal Welfare and Ethics Review Board (AWERB) of the University of Aberdeen. Animal work was conducted under Dr Wenlong Huang's UK Home Office Project License (PPL: P0E0BDEA1).

Because studies on CNS repair require a mimic of the in vivo microenvironment and cellular connections, primary neuron cultures were used here. Our assumption was that if hydrogels were successful in promoting growth in a primary neuron culture model, they could be used to study the pathogenesis of peripheral nerve injuries and CNS diseases, as well as in regenerative medicine, an emerging field of spinal cord injury research [18].

The cortical neurons were cultured according to Beaudoin et al. [11], with the modification that postnatal day 0–1 Sprague Dawley rat cortices were used instead of mouse cortices. The detailed isolation, dissociation, plating and culture procedures used in the present study are described below. Sprague Dawley rats at postnatal days 0–1 (mixed sexes) were culled by cervical dislocation and decapitated with large sterile scissors. The skin was meticulously excised using scissors, followed by the creation of an incision running parallel to the sagittal suture. The skull covering the cortex was crushed laterally, starting from the midline of the parietal bones toward the frontal bones, using Dumont #5 forceps. Upon exposing the entire cortex, the hindbrain was carefully extracted and transferred to a 60 mm Petri dish containing HBSS (Invitrogen, UK). Using a dissecting microscope, the midbrain was meticulously excised, leaving the cortex entirely isolated. The meninges were then delicately removed while preserving the integrity of the cortex. Subsequently, the midbrain hippocampus was removed, and the cortex was then sectioned into smaller pieces and transferred into a 15 mL centrifuge tube (Corning, UK). The tissue pieces were dissociated enzymatically with a mixture of 10 U/mL papain in Retinal Buffer, triturated gently with a P1000 pipette, and left to incubate at 37°C for 30 min in a CO_2_ incubator. The solution was shaken after 15 min to allow complete digestion. The enzymatic process was stopped by adding 3 mL of culture medium containing 10% foetal bovine serum, and the tissue was further triturated up to 3 times using a P1000 pipette and centrifuged at 353 × g (ALC Multispeed Refrigerated Centrifuge PK 121R, ALC International, UK) for 10 min. The supernatant was removed, and the cell pellet was resuspended in 1 mL of complete Neurobasal medium, included B‐27 Supplement (50X, 2%, Gibco, UK), Glutamax (1%; Thermo‐Fisher scientific, UK) and Penicillin‐Streptomycin (1%; Sigma, UK) with Neurobasal‐A media (Thermo‐Fisher scientific, UK). Then filtered through a 70 µm cell strainer (10788201, Fisher Scientific, UK) and the single cell numbers were counted using a hemocytometer. The final cell concentration was adjusted to 70,000 cells/mL by diluting with complete Neurobasal medium, and the cortical neurons were plated at densities of 70 000 cells/mL on 10 µg/mL poly‐d‐lysine (PDL) coated round 18 mm glass coverslips or 3.1 × 10^4^ cells/cm^2^ on 1% silk gel circles in 8‐well chambers (177402PK, Thermo Scientific, UK). The cells were cultured for up to 48 h at 37°C in the incubator.

### 2.2 Silk‐Based Hydrogels

*B. mori* cocoons were processed as detailed previously [19]. Briefly, cocoons were cut into approx. 5 mm × 5 mm pieces and boiled in 0.02 M sodium carbonate solution for 1 h [19, 20, 21]. The fibers were then thoroughly washed in dH_2_O, manually compressed to remove excess liquid, carefully separated, and air dried in a fume hood.

The silk hydrogel was prepared following a published protocol [20]. The degummed *B. mori* silk fibers were dissolved in 9.3 M LiBr solution at 60°C for 4 h to obtain a silk fibroin solution. This was followed by 48 h dialysis against dH_2_O using Slide‐A‐Lyzer G2 dialysis cassettes (3.5K MWCO, 87725, Thermo Fisher, UK), with periodic water changes. The solution was then centrifuged at 9500 × *g* (Avanti JXN‐26 Series High‐Speed Centrifuge, Beckman Coulter, UK) for 20 min to remove impurities and silk aggregates formed during dialysis. The final silk solution concentration was determined gravimetrically [19]. The silk solution was filter‐sterilized by passage through a 0.22 mm syringe filter and stored in a fridge at 4°C. The silk solution was diluted to 1% w/v with dH_2_O one day before the experiment.

The solution–gel transition was initiated using a probe sonicator (MSE 11–73 Ultrasonic Disintegrator, MSE, UK). For 2D cortical neuron experiments, the silk hydrogels were prepared using two sonication cycles: sonication for 30 s at room temperature at medium power, followed by a 30 s off cycle on ice. A 100 µL sample of 1% w/v sonicated silk undergoing the solution–gel transition was dispensed into an 8‐well chamber slide (177402, Thermo Fisher, UK). The solution–gel transition was allowed to complete overnight at 37°C in a 5% CO2 / 95% air incubator (NU‐581DE; Nuaire).

For 3D cortical neuron experiments, the solution–gel transition was initiated as above. Cells were added to the silk solution 30 min after the sonication cycle. Based on pilot optimization experiments, a final cell density of 200 000 cells/mL silk hydrogel solution was used for the 3D cultures.

### 2.3 Treatment With Epac2 Agonist S‐220

S‐220 was used for specific Epac2 activation. S‐220 is a specific non‐hydrolysable analogue that potently and selectively activates Epac2 over Epac1 both in vitro and in vivo [22].

In the conventional cortical neuron cultures grown on PDL‐coated coverslips, S‐220 was added to the complete Neurobasal medium at final concentrations of 1.25, 2.5, and 5 µm.

For 2D cortical neuron cultures grown on 1% silk hydrogels, S‐220 was applied using two different approaches. First, S‐220 was added directly to the complete Neurobasal medium at final concentrations of 2.5 or 5 µM after hydrogel formation. Second, S‐220 was incorporated into the 1% silk solution before completion of the solution–gel transition, resulting in a final concentration of 5 µm S‐220 within the hydrogel.

No S‐220 was added to the 3D silk hydrogel cultures, which were used to establish and characterize the 3D cortical neuron culture model.

### 2.4 Immunocytochemistry

Cells were fixed with ice‐cold, freshly prepared 4% paraformaldehyde (PFA) in phosphate‐buffered saline (PBS) for 30 min at room temperature, followed by three 10 min washes with PBS. Non‐specific binding was blocked with 10% goat serum (Sigma, UK) in PBS containing 0.2% Triton X‐100 (Sigma, UK) and 0.1% sodium azide (Sigma, UK) for 1 h at room temperature. Samples were then incubated overnight at 4°C with mouse anti‐β‐tubulin III antibody (1:1000, T8578, Sigma‐Aldrich, UK). After three PBS washes, samples were incubated with Alexa Fluor 488 anti‐mouse secondary antibody (1:400, AB_2534069, Invitrogen, UK) for 2 h at room temperature.

For 3D silk hydrogel cultures, samples were fixed with ice‐cold 4% PFA for 30 min and washed three times with PBS. To improve antibody penetration, samples were blocked and permeabilized for 1 h at room temperature using 10% goat serum in permeabilization buffer containing 10.3 g sucrose, 0.292 g NaCl, 0.06 g MgCl_2_, 0.476 g HEPES and 0.5 mL Triton X‐100 in 100 mL 1× PBS, adjusted to pH 7.2. Samples were then incubated overnight at 4°C with mouse anti‐β‐tubulin III antibody (1:1000). After three washes with 0.5% Tween‐20 in PBS, samples were incubated with Alexa Fluor 488 anti‐mouse secondary antibody (1:400) for 3 h at room temperature and then washed three times with 0.5% Tween‐20 in PBS.

For all experiments, nuclei were counterstained with Hoechst 33342 (2 µg/mL in PBS; Sigma, UK) for 5 min at room temperature. Samples were then washed three times with distilled water. Coverslips were mounted on microscope slides with PBS/glycerol mounting medium at a 1:8 ratio, sealed with nail polish, and stored at 4°C until imaging.

### 2.5 Randomization and Blinding

Cultures were randomized to treatment groups, with the plate position balanced across conditions. Although the researcher was aware of allocation during injury and treatment, all imaging and quantitative analyses were conducted on anonymized, coded datasets with a randomized acquisition order and the same standard image acquisition settings for all images. Unblinding occurred only after the final statistical analysis had been performed.

### 2.6 Imaging

**FIGURE 1:**
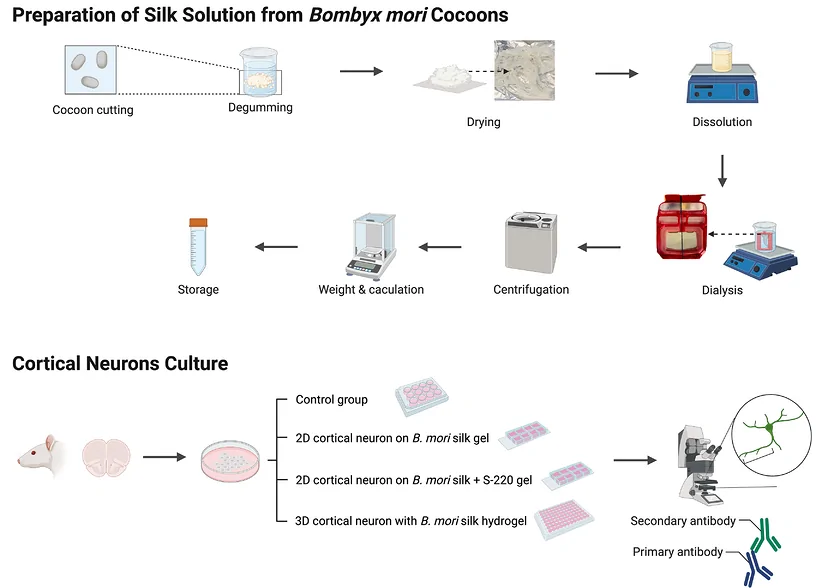
Experimental workflow for silk hydrogel preparation and cortical neuron culture. *B. mori* cocoons were cut into small pieces, degummed in sodium carbonate solution, washed, dried, dissolved in lithium bromide, dialyzed against distilled water, centrifuged to remove impurities, filter sterilized, sonicated to initiate gelation, and used to prepare 1% silk hydrogels. Cortical neurons were then cultured on PDL coated coverslips, 2D silk hydrogels or embedded within 3D silk hydrogels, with or without S‐220 treatment, before immunocytochemical staining, imaging, and neurite outgrowth analysis.

An Eclipse Ti‐E fluorescence microscope (Ti‐E, Nikon Instruments Europe B.V., UK) was used to collect all images. Images were captured using a Nikon‐DS‐U3 camera with 20X objective magnification (CFI S Plan Fluor ELWD 20XC, Nikon Instruments Europe B.V., UK) and NIS‐elements 5.1.1 software (Nikon Instruments Europe B.V., UK). The images were saved in ND2 format, with files containing all image information. Where applicable, fluorescence images were merged using Fiji/ImageJ2 (version 2.14.0/1.54f). The experimental workflow diagram shown in Figure 1 was created using BioRender.com.

### 2.7 Quantification

CSIRO HCA‐Vision Software (hca‐vision.csiro.au) was used to quantify the neurite length. Two main measures of neurite outgrowth were used: total neurite length (TNL) and longest neurite length (LNL). For each neuron, TNL was defined as the sum of the lengths of all neurite branches associated with that neuron. LNL was defined as the length of the single longest neurite branch associated with the same neuron. TNL provides a measure of overall neurite elaboration, incorporating the contribution of all neurite branches, whereas LNL reflects the maximum extent of neurite elongation. Measurements from all neurons within each field of view were averaged, and data from multiple non‐overlapping fields were subsequently combined within each biological replicate. Individual neurons and fields of view were not treated as independent biological replicates.

For morphological analysis, neurons in each image were divided into four groups based on a previous method [23, 24] according to the following categories: complex, simple, growth cone and no growth cone. Neurons were classified as ‘complex’ if primary and secondary axons were present and/or the LNL was greater than 10 times the diameter of the cell body. In the ‘simple’ category, neurons have only primary neurites, whose lengths are less than 10 times the diameter of the cell body. Neurons show only a short growth cone in the ‘growth cone’ category. In the ‘none’ category, only the cell body is present (Figure 4A).

When calculating the number of branches per neuron, projections that were longer than the diameter of the cell body were scored as branches [25].

### 2.8 Statistical Analyses

All experiments were performed as three independent experimental repeats (*N* = 3). Within each independent experimental repeat, cortical neuron cultures were prepared from 3–5 independent postnatal rat‐derived samples, which were considered biological replicates (*n* = 3–5). In this study, N refers to independent experimental repeats, while n refers to independent biological replicates within each repeat. Multiple non‐overlapping images/fields of view were acquired from each culture for neurite quantification, but these images/fields of view were not treated as independent replicates.

Data are presented as the mean ± standard deviation (SD). Statistical analyses were performed using GraphPad Prism 9 (GraphPad Software Inc., California, US). The Shapiro–Wilk normality test was performed to check for normal distribution of the data and suitability of parametric tests; normality was met with all the data. For comparisons between two groups, unpaired *t* test was used. For comparisons among multiple groups, one‐way ANOVA with Dunnett *post hoc* analysis was used. In all figures, statistically significant differences were indicated as *p* < 0.05 (*), *p* < 0.01 (**), *p* < 0.001 (***).

## 3 Results

### 3.1 Epac2 Activation by S‐220–Enhanced Cortical Neuron Outgrowth in In Vitro Cell Culture

To verify the optimal concentration of S‐220, cultured cortical neurons were exposed to S‐220 concentrations of 1.25, 2.5, and 5 µm. Treatment with 1.25 µm S‐220 had no significant effect on the mean LNL, although it produced a significant increase in the mean TNL. However, 2.5 and 5 µM progressively produced more significant increases in both TNL and LNL.

**FIGURE 2:**
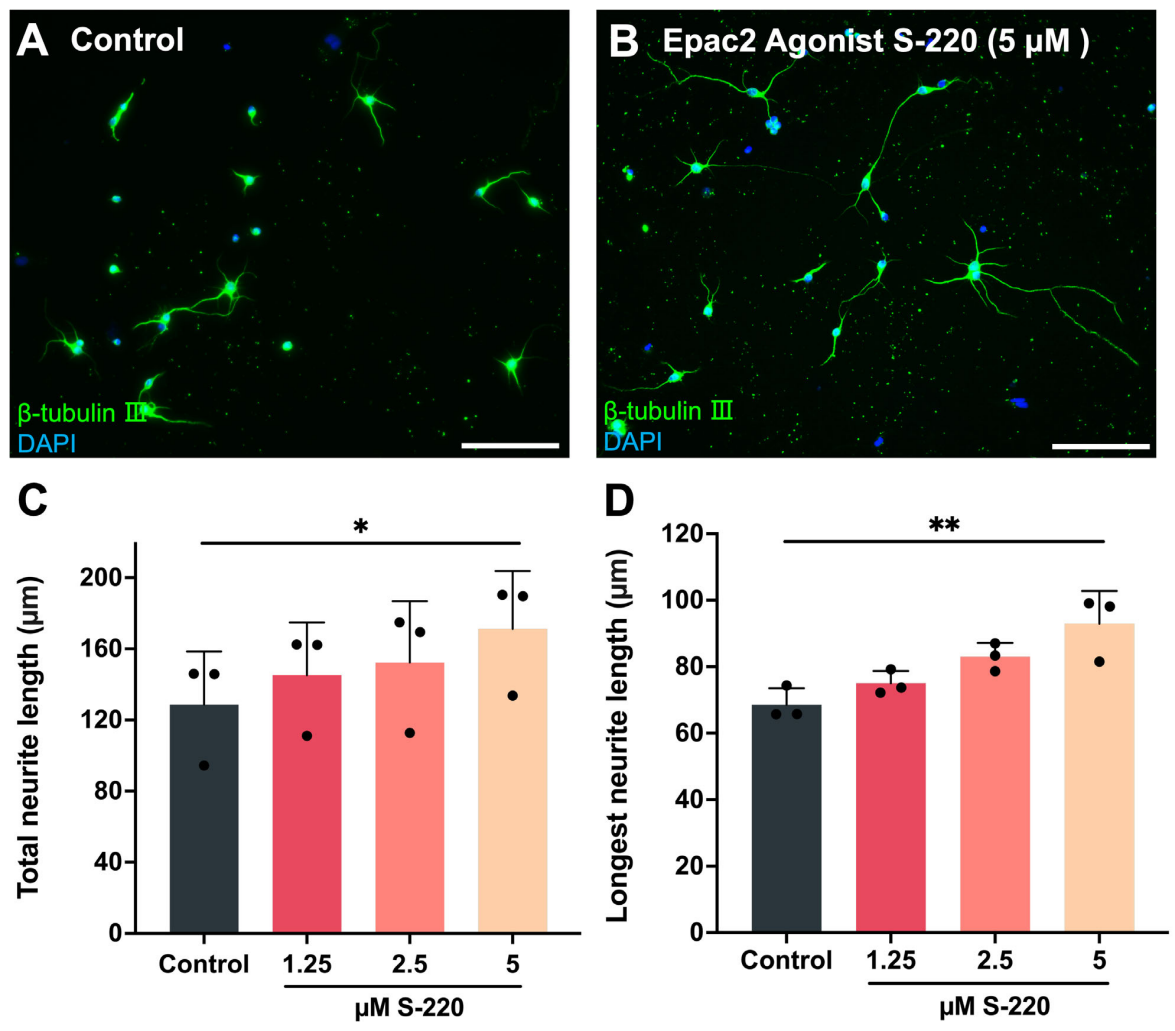
Characterization of outgrowth of cortical neuron neurites cultured on PDL‐coated coverslips with different concentration Epac2 agonist S‐220 in culture media. Representative fluorescence images of neurons cultured in (A) the control condition and (B) cultured on PDL‐coated coverslips with 5 µM S‐220 in culture media (The cultures were grown for 48 h. Treatments were applied in the third hour). (C) Total neurite length. D, Longest neurite length. Cultures were kept for 48 h. Circles represent independent experiments (*N* = 3). Scale bar: 100 µm.

S‐220 at 2.5 µM produced a significant increase in the mean TNL when compared to the controls (Figure 2C). S‐220 at 2.5 µM also showed a significant enhancement of the mean LNL.

Both the mean TNL and mean LNL were significantly increased over the control values by treatment with 5 µM S‐220 for 48 h (Figure 2C,D). When compared with the lower concentration treatment groups, the 5 µM S‐220 treatment group showed a significant enhancement of neurite outgrowth (Figure 2C,D). The increase in TNL reflected greater overall neurite elaboration, based on the combined lengths of all neurite branches associated with each neuron, whereas the increase in LNL reflected greater maximal extension of the longest individual neurite branch.

Examination of the effects of S‐220 in terms of morphological changes (Figure 4B) revealed that cultures treated with 5 µM S‐220 showed the highest percentage of total neurons with complex morphology. Neurons in control cultures showed the highest percentages of cells with ‘no growth’, ‘growth cone’ and ‘simple morphology’ compared to the cells in the 5 µM S‐220 group. The percentages of ‘simple’, ‘growth cone’ and ‘no growth’ neurons decreased with increasing S‐220 concentrations.

### 3.2 Effects of the Silk Hydrogel–S‐220 Combination on Cortical Neuron Growth

#### 3.2.1 Effects of the Silk Hydrogel Alone

**FIGURE 3:**
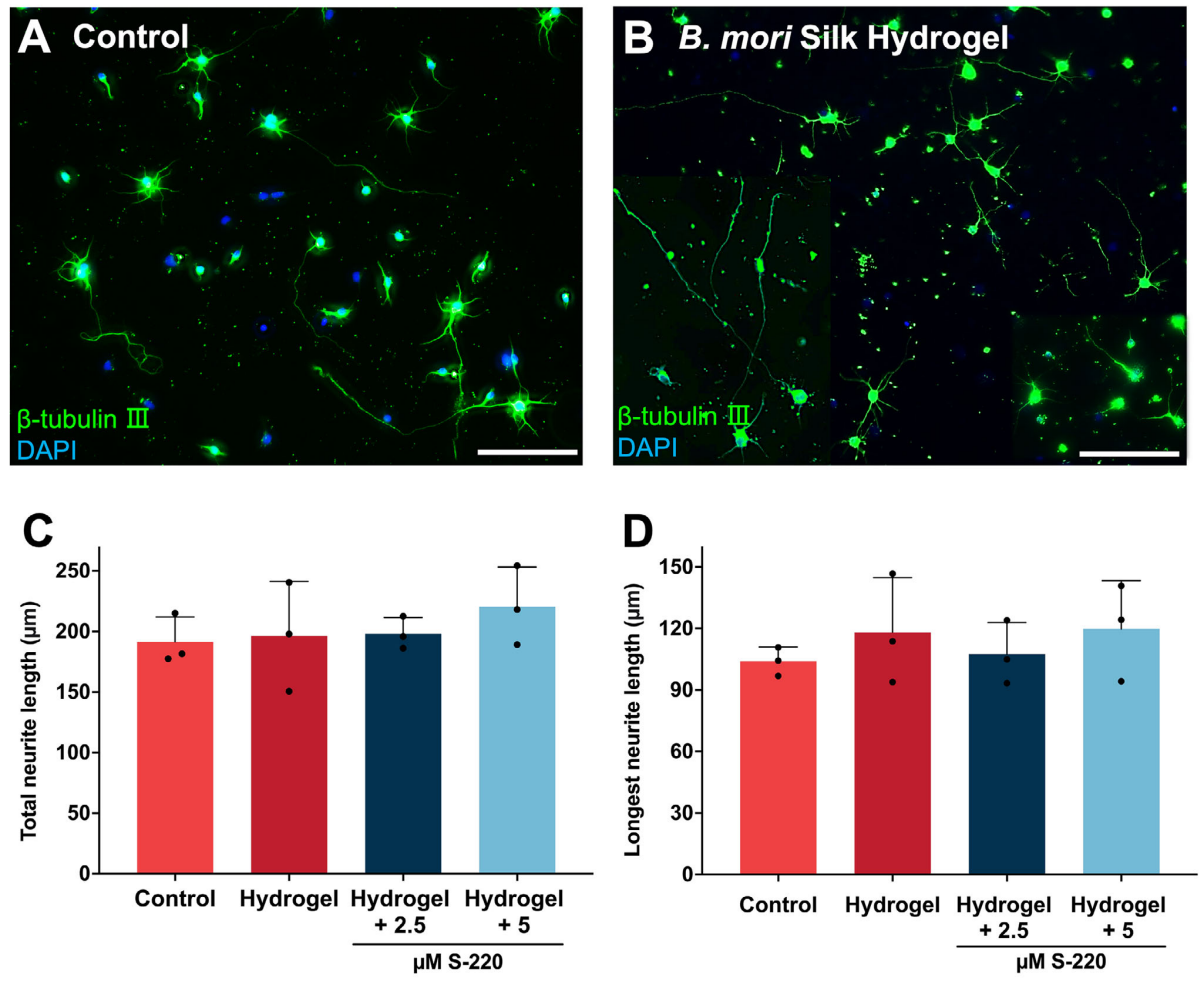
Characterization of outgrowth in 2D cortical neurons cultures grown on 1% silk hydrogels with or without Epac2 agonist S‐220 added to the culture medium. Representative fluorescence images of neurons cultured under (A) control condition and (B) on 1% silk hydrogels. (C) Total neurite length. (D) Longest neurite length. Cultures were grown for 48 h. Circles represent independent experiments (*N* = 3). Scale bar: 100 µm.

The neurite outgrowth on 1% silk hydrogel was similar to that of the control group cultured on PDL‐coated coverslips (Figure 3C,D). However, the mean TNL was increased slightly above that of the control group when cells were grown on 1% silk hydrogel with 5 µM S‐220.

**FIGURE 4:**
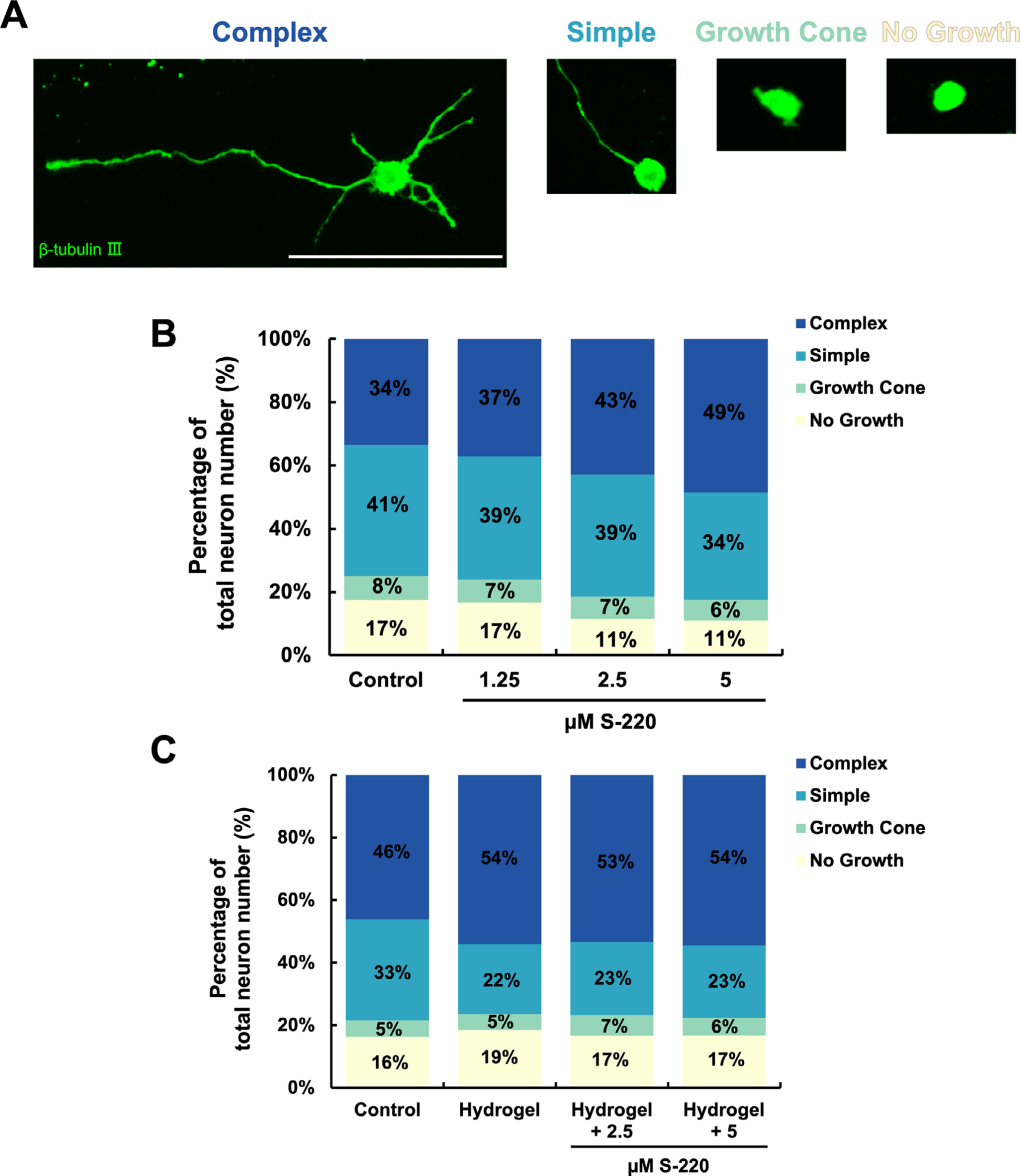
Effects of different concentrations of S‐220 and 2D culture substrates on cortical neuron morphology. A, Representative images showing classification of neuronal morphology. (B, C) Data are presented for the mean percentage of total cell numbers showing no growth, a small growth cone, simple morphology or complex morphology. Circles represent independent experiments (*N* = 3). Scale bar: 100 µm.

Morphological analysis further supported the effect of S‐220 on cortical neuron development (Figure 4B). In cultures grown on PDL‐coated coverslips, treatment with 5 µM S‐220 produced the highest proportion of neurons with complex morphology, whereas control cultures showed higher proportions of neurons classified as no growth, growth cone, or simple morphology. The proportion of neurons with less developed morphology decreased with increasing S‐220 concentration.

In 2D silk hydrogel cultures, analysis of the effects of the silk hydrogel with or without S‐220 on cell morphology (Figure 4C) revealed that 1% hydrogel + 2.5 µM S‐220 produced the highest percentage of neurons with growth cone morphology, whereas 5 µM S‐220 produced the highest percentage of neurons with complex morphology. The percentage of neurons showing ‘no growth’ morphology was decreased when compared with the group grown on 1% hydrogel alone.

#### 3.2.2 Impact of Silk Hydrogels With and Without S‐220

The impact of S‐220 in combination with silk hydrogel was examined by preparing a 1% silk hydrogel that were loaded with 5 µM S‐220 rather than simply added to the culture medium.

**FIGURE 5:**
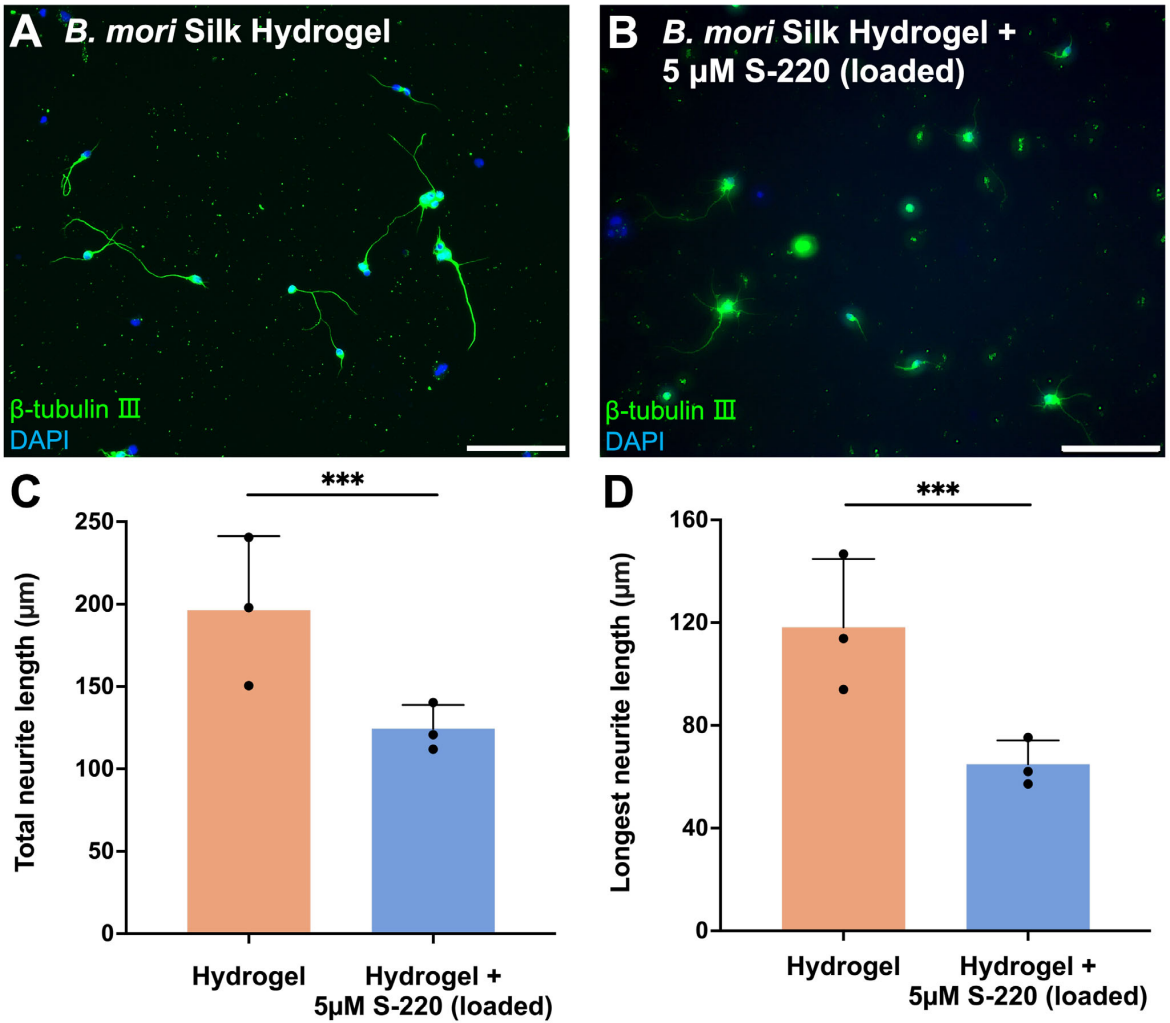
Characterization of outgrowth of cortical neurons cultured in 2D on 1% silk hydrogels loaded with Epac2 agonist S‐220. Representative fluorescence images of neurons cultured (A) on 1% silk hydrogels and (B) in the presence when loaded with 5 µM S‐220. (C) Total neurite length. (D) Longest neurite length. Cultures were grown for 48 h. Circles represent independent experiments (*N* = 3). Scale bar: 100 µm.

Incorporating 5 µM S‐220 into the hydrogel significantly decreased the mean TNL (Figure 5). The mean LNL was reduced in the group treated with 1% hydrogel + 5 µM S‐220 (no morphological analysis was conducted). Overall, the combined method showed that the TNL and LNL outcomes differed between the group treated with the silk hydrogel loaded with 5 µM S‐220 vs the group treated with culture medium containing added S‐220 (Figure 3).

### 3.3 The Establishment of a 3D Cortical Neuron Model Using the Silk Hydrogel

**FIGURE 6:**
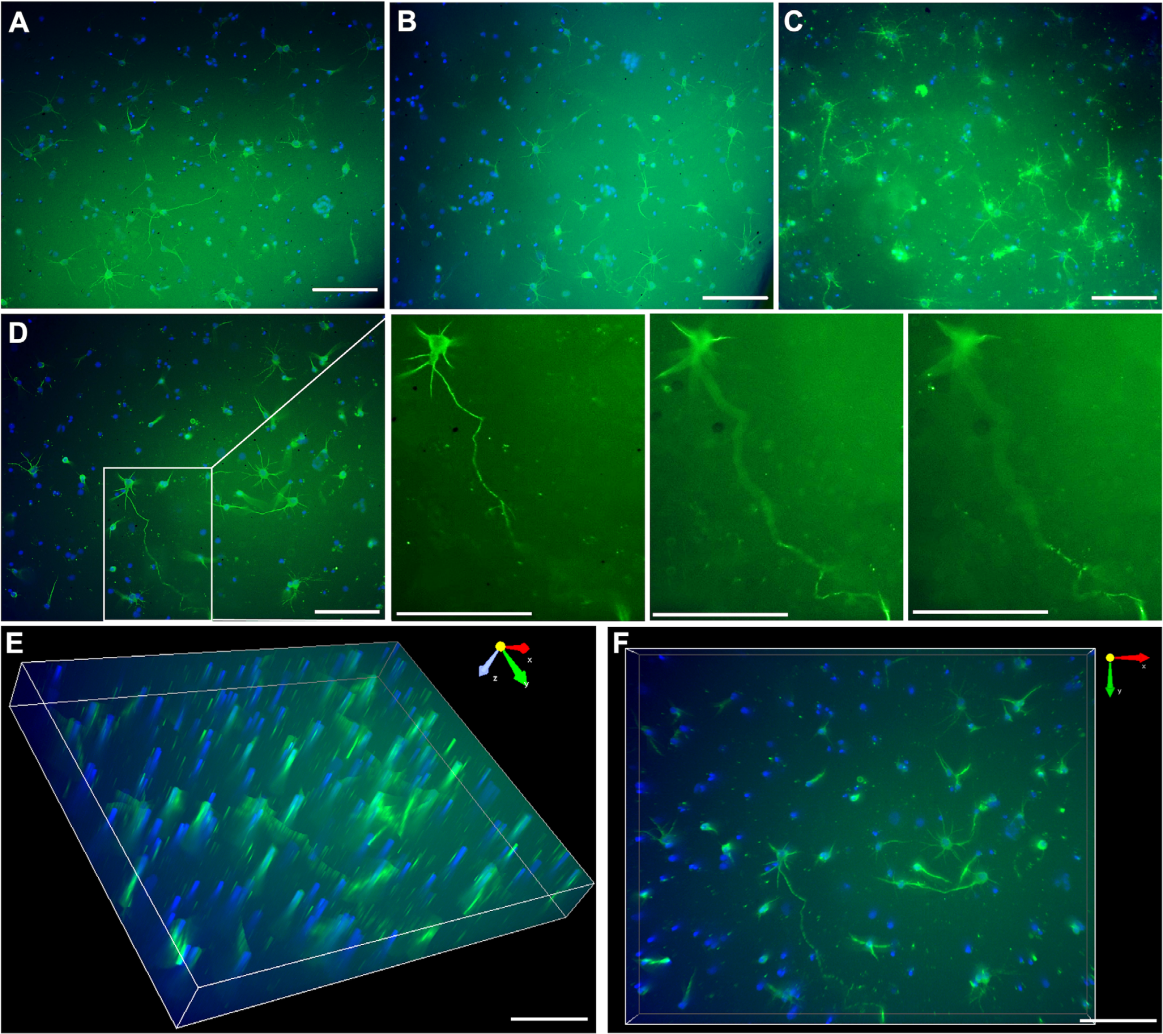
Establishing cortical neurons 3D cultures with silk hydrogel. (A) Representative fluorescence images of cortical neurons cultured with silk hydrogels at the density of 325 000 cells/mL. (B) Representative fluorescence images of cortical neurons cultured with silk hydrogels at the density of 350 000 cells/mL. (C) Representative fluorescence images of cortical neurons cultured with silk hydrogels at the density of 400 000 cells/mL. (D) Representative fluorescence images of cortical neurons cultured with silk hydrogels at the density of 200 000 cells/mL, with zoomed maximal intensity projections of Z‐stack images. (E, F) Showing different rotations in the Z planes of the cortical neurons shown in D. Cultures were grown for 72 h. Scale bar: 100 µm.

Silk hydrogels were tested for their ability to support 3D growth of postnatal rat cortical neurons (Figure 6). The cell seeding densities were first optimized by trialing cell concentrations of 325 000, 350 000, and 400 000 neurons/mL, but the fluorescent staining results indicated that these cell densities were too high. The lengths of the neurites were longer in the group with a cell density of 200 000 neurons/mL than in the groups with higher cell densities (Figure 6D), suggesting that neurons at too high a density cannot obtain sufficient nutrients to support growth (Figure 6A–C). For this reason, a cell density of 200 000 neurons/mL was deemed optimal (Figure 6D).

The hydrogel also supported 3D neurite outgrowth of cortical neurons, as fluorescence microscopy using different rotational planes and focuses revealed neurites growing in all directions other than just horizontal (Figure 6E).

**FIGURE 7:**
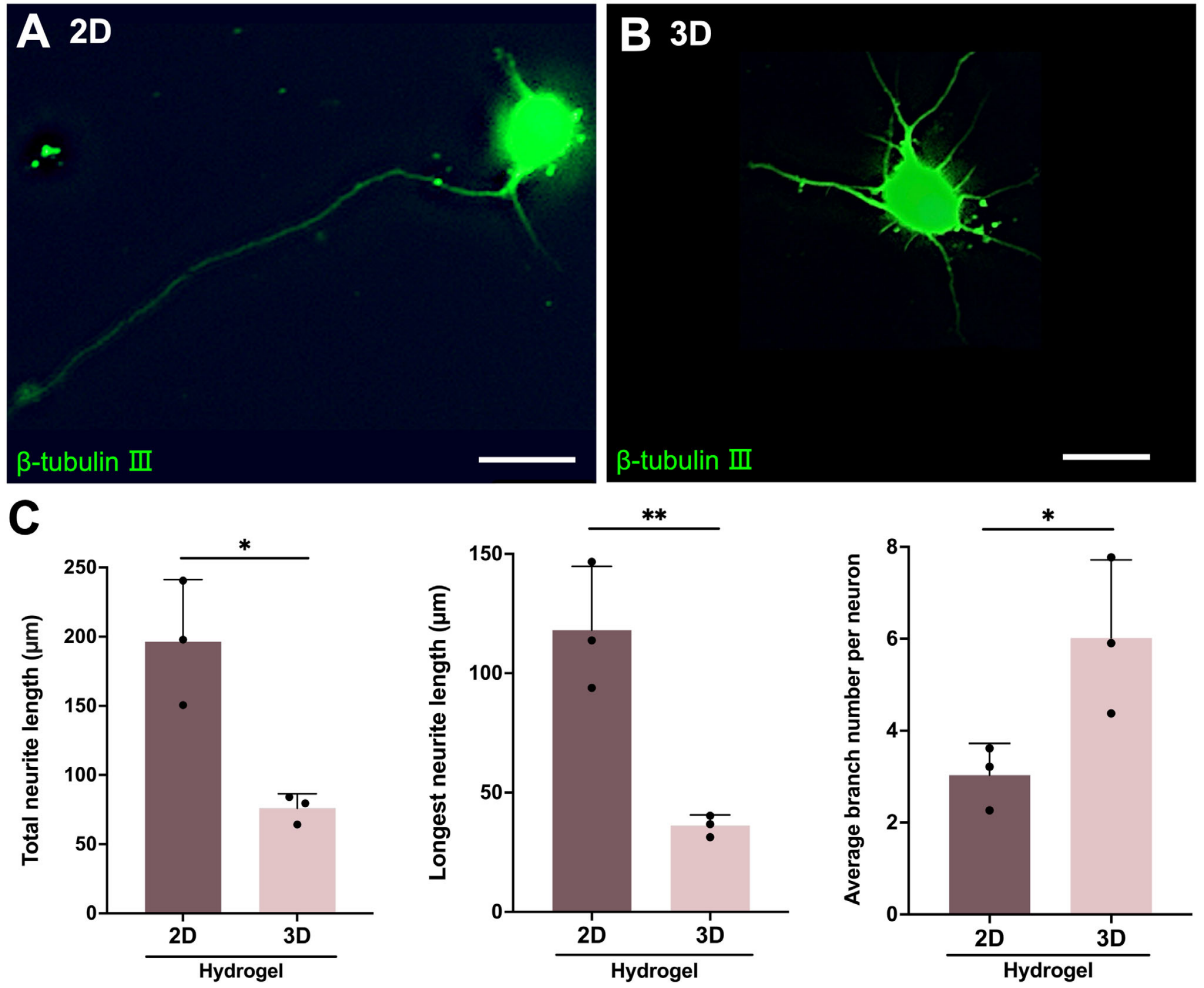
Comparison with 2D cultures reveals that 3D cultures promote more complex branching. (A) Representative fluorescence images of the branches in cortical neurons in the 2D 1% silk hydrogel group. (B) Representative fluorescence images of the branches in the 3D culture group. (C) Quantification of the total neurite length, longest neurite length, and branch numbers. Circles represent independent experiments (*N* = 3). Scale bar: 20 µm.

Quantitative analyses showed that the hydrogel supported the 3D outgrowth of cortical neurons, although the mean TNL and LNL were decreased when compared with those observed in the 2D cultures (Figure 7C). However, further quantification showed that the cortical neurons had 50% more primary branches when grown in 3D cultures versus 2D cultures (Figure 7C). Overall, the results suggest that silk hydrogels have the capability to support excellent in vitro neurite growth (in both 2D and 3D cultures).

## 4 Discussion

We demonstrated that enhancing Epac2 effects using the selective agonist S‐220 significantly increased neurite outgrowth from cultured postnatal cortical neurons. Epac2 activation also increased neurite outgrowth in cortical neurons cultured on a silk hydrogel. In addition, we established a silk hydrogel‐based 3D postnatal cortical neuron culture model and showed that this system supported multidirectional neurite extension and increased branching complexity compared with conventional 2D cultures.

The present study used a simplified 1% *B. mori* silk hydrogel without additional collagen or extracellular matrix functionalization. This reductionist system allowed us to examine how the silk hydrogel environment, alone or in combination with Epac2 activation, influenced neurite outgrowth and branching morphology in postnatal cortical neurons. Unlike previously reported silk‐based cortical culture systems incorporating collagen or other extracellular matrix components, the present model was designed to examine neuronal responses to the silk hydrogel itself [26, 27]. The 3D cultures supported multidirectional neurite extension and increased branching complexity, although total and longest neurite lengths were lower than those observed under 2D culture conditions. These findings suggest that culture dimensionality and hydrogel architecture may differentially influence neurite elongation and branching.

The in vitro cortical neuron analysis showed that silk hydrogel supported the growth of postnatal cortical neurons, further supporting its biocompatibility and suitability as a neural culture substrate, as previously reported [28]. Beyond supporting neuronal survival and outgrowth in vitro, these findings motivate further evaluation of silk hydrogels for CNS repair related applications. Both our work and studies from other groups indicate that the storage modulus (G′) of silk hydrogels can fall within the range reported for adult rat brain tissue [29] and human cortex tissue [30], suggesting that 1% silk hydrogel can approximate the apparent stiffness of the CNS microenvironment. However, stiffness alone is not sufficient to recapitulate neural tissue mechanics. Brain and spinal cord tissues show pronounced viscoelastic and time‐dependent behavior, and material parameters such as loss modulus (G″), damping (tan δ) and stress relaxation are also expected to influence cell matrix mechanotransduction and host tissue responses following implantation. In the context of silk hydrogels, recent work has highlighted the importance of considering these viscoelastic features, rather than G′ alone, when designing silk‐based matrices for neural applications [31], as reviewed in [32]. Consistent with this broader biomechanical view, prior histological analyses in neuronal culture suggested that 1% silk hydrogel with an elastic stiffness on the order of approximately 100 Pa promoted favorable cortical neuron growth [28]. Moreover, in vivo studies support the translational feasibility of injectable silk hydrogels. Silk hydrogels delivered by intrastriatal injection were reported to be biocompatible within mouse brain tissue [33], and silk hydrogels used as injectable fillers for rat stroke cavities showed stability, effective space filling and sustained biocompatibility over 7 weeks and 12 months, including interactions with glial scars and support of endogenous cell proliferation [34, 35]. Collectively, these findings support silk hydrogels as promising biomaterials for CNS repair, while emphasizing that both elastic and viscoelastic tissue behaviors should be considered when optimizing materials for injectable neural applications.

The successful culture of cortical neurons within silk hydrogels supports the use of this system as an in vitro platform for investigating neurite outgrowth, neuronal morphology and neuron–biomaterial interactions relevant to CNS repair research. Neurons are highly polarized cells with specialized axonal and dendritic compartments that support synaptic connectivity and information processing [36, 37]. However, neuronal morphology can vary depending on neuronal subtype and experimental conditions, and multiple axon phenotypes with functional axon initial segments have also been reported [38].

In the present study, our finding of significant increases in the branch numbers in the 3D cortical neuron models indicated that silk hydrogels support neuron development. Thus, primary neuron cultures were transformed into 3D models with enhanced neurite complexity. The increased branching observed in the 3D silk hydrogel cultures is likely to reflect the combined influence of dimensionality, matrix architecture and cell material interactions. In 2D cultures, neurites extend primarily along a flat substrate, whereas in 3D hydrogels they can extend through a spatially complex matrix in multiple directions. This additional dimensionality may support more complex branching patterns. At the same time, the hydrogel network may physically and topographically constrain long, linear neurite extension, leading to altered growth trajectories and increased branching rather than longer unidirectional neurite elongation. This interpretation is consistent with previous observations that substrate architecture can influence neurite extension and branching behavior [39]. In addition, unmodified *B. mori* silk does not provide specific cell adhesive motifs for direct integrin mediated cell matrix interactions [40], which may partly limit neurite extension within the 3D matrix. One strategy to address this limitation would be to functionalize silk with extracellular matrix mimetic motifs, such as arginine‐glycine‐aspartic acid (RGD) or glycine‐arginine‐glycine‐aspartic acid (GRGD), to improve integrin engagement and better mimic aspects of the native extracellular matrix environment [41, 42]. Future advances in 3D bioprinting and hydrogel functionalization may further improve the spatial organization and biological performance of silk‐based neural culture systems [43].

An important observation in the present study was that adding S‐220 to the culture medium enhanced neurite outgrowth, whereas incorporating S‐220 directly into the silk hydrogel did not produce the same pro‐growth effect. The reason for this difference is not yet clear. Possible explanations include altered S‐220 availability, restricted diffusion, interaction of S‐220 with the silk hydrogel network, or differences in the timing and duration of neuronal exposure to the agonist. Because the present study did not directly assess S‐220 release kinetics or local concentration within the hydrogel, these possibilities remain speculative. Future studies should therefore include release profiling, dose optimization and time course analyses to determine whether controlled delivery of S‐220 from silk hydrogels can be optimized to support neurite extension.

In the case of Epac, its roles in physiology and pathophysiology have been well established over the past 20 years [44]. Because Epac orchestrates signaling events that regulate important neuronal functions, a reasonable assumption is that defects in Epac genes, such as mutations or pathophysiological changes in Epac signaling, may underlie certain neurological disorders [45, 46, 47]. The use of various research tools has also shown that Epac is involved in a variety of neuronal functions, including neurotransmitter release [48], neurite outgrowth, and neuronal/glial differentiation [49, 50]. Shewan et al. demonstrated that siRNA knockdown of Epac1 and Epac2 in adult dorsal root ganglion neurons significantly reduced neurite outgrowth [51]. However, since adult neurons predominantly express Epac2, rather than Epac1, neurite outgrowth seems likely to be mediated by Epac2 [16].

Previous work highlighting PKA and Epac indicates that these proteins can play opposite roles in axonal regrowth. We have previously demonstrated that spiking culture medium with S‐220 significantly increased axonal regrowth in an ex vivo model of spinal cord injury [16], thereby corroborating the observations that Epac2 is a general agonist, whereas S‐220 is a specific agonist. We also found S‐220 activated Epac2, protected neurons and oligodendrocytes after injury, reduced gliosis, and boosted axon growth into the lesion gap in the in vitro cortical mechanical injury model [52]. In the present work, S‐220 at 5 µM significantly promoted neurite outgrowth in cultured cortical neurons, further supporting a pro‐growth effect of Epac2 activation in this experimental system. However, Epac2 mediated neurite outgrowth is unlikely to be explained by a single linear pathway. Past reviews have summarized the complex mechanisms through which Epac2 signaling may influence neuronal morphology and neurite extension [17, 53]. Epac2 activates Rap1 dependent signaling, which may subsequently influence downstream pathways including, including, which subsequently regulates the Protein Kinase B (AKT) [54, 55] and B‐Raf‐MER‐ERK [56, 57, 58] signaling pathways. These pathways are known to contribute to axon growth, guidance and neuronal morphological regulation [59]. In addition, Epac2 dependent responses may interact with other cAMP‐regulated mechanisms, including PKA‐dependent signaling, making the final cellular outcome highly context dependent.

Although our findings show that S‐220 enhanced neurite outgrowth in cortical neuron cultures, the present study did not directly measure downstream Epac2 signaling events, such as Rap1, AKT, ERK or other pathway activation. Therefore, these results should be interpreted as morphological evidence supporting a pro‐growth effect of S‐220/Epac2 activation in this culture model, rather than direct molecular confirmation of the full signaling pathway. Future studies using pathway specific inhibition, genetic manipulation, or biochemical assays will be required to define the downstream mechanisms by which Epac2 activation promotes neurite outgrowth in silk hydrogel‐based cortical neuron cultures. It will also be important to determine whether the morphological changes observed in the present model correspond to functional neuronal maturation, for example by assessing synaptic marker expression, calcium activity or electrophysiological properties.

Taken together, the present findings establish 1% silk hydrogels as a permissive substrate for cortical neuron culture in both 2D and 3D. Selective Epac2 activation enhanced neurite outgrowth when S‐220 was supplied in the culture medium, whereas incorporation of S‐220 directly into the hydrogel did not produce the same effect. The 3D hydrogel system supported multidirectional neurite extension and increased branching complexity, despite reductions in total and longest neurite lengths compared with 2D cultures. The findings establish the system as a useful in vitro platform for investigating neuronal responses to biomaterial environments and for comparing different approaches to bioactive compound delivery. Further work should focus on hydrogel functionalization, characterization of S‐220 release and evaluation of optimized conditions in more complex neural and injury‐relevant models.

## 5 Conclusions

Overall, this study establishes the use of a silk hydrogel‐based cortical neuron culture platform, together with selective Epac2 activation by S‐220, as an in vitro approach for investigating neurite outgrowth and neuron biomaterial interactions. The addition of S‐220 to the culture medium enhanced neurite outgrowth in cortical neuron cultures, whereas incorporation of S‐220 directly into the hydrogel did not produce the same pro‐growth effect. Compared with conventional 2D cultures, the 3D silk hydrogel culture model allowed cortical neurites to extend in multiple directions and was associated with increased branching complexity, although overall neurite length was reduced. Together, these findings demonstrate the value of this system as an in vitro platform for evaluating neuronal responses to silk hydrogel environments and for optimizing the delivery of bioactive compounds relevant to neural repair research.

## Author Contributions

H.M.: conceptualization, data curation, formal analysis, funding acquisition, investigation, methodology, project administration, resources, software, validation, visualization, writing – original draft, writing – review & editing; F.P.S.: training, methodology, resources, writing – review & editing; W.H.: conceptualization, data curation, funding acquisition, methodology, project administration, resources, supervision, validation, visualization, writing – review & editing; All authors reviewed and approved the final manuscript.

## Funding

P.S. acknowledges support from a DFG Heisenberg Grant (SE 3307/1‐1) and funding from the Free State of Thuringia (Thüringer Aufbaubank) and European Fonds for Regional Development (EFRE) with grant no. 2024FGI0005.

## Conflicts of Interest

The authors declare no conflicts of interest.

## Data Availability

All data created during this research are openly available from the University of Aberdeen‐Pure, DOI: 10.20392/998299fe‐e43e‐47a3‐b31e‐040749664020, at https://abdn.elsevierpure.com/en/datasets/data‐for‐investigating‐the‐effects‐of‐the‐combination‐of‐bms‐hydr/.
